# Educational Videos Versus Question Banks: Maximizing Medical Student Performance on the United States Medical Licensing Examination Step 1 Exam

**DOI:** 10.7759/cureus.38110

**Published:** 2023-04-25

**Authors:** Karina R Clemmons, Jasna Vuk, Diane M Jarrett

**Affiliations:** 1 Department of Medical Humanities and Bioethics, University of Arkansas for Medical Sciences, Little Rock, USA; 2 Academic Affairs, Educational and Student Success Center, University of Arkansas for Medical Sciences, Little Rock, USA; 3 Department of Family and Preventive Medicine, University of Arkansas for Medical Sciences, Little Rock, USA

**Keywords:** question banks, educational videos, retrieval practice, active study methods, medical education

## Abstract

Objective

The aim of this research was to determine if medical students' use of the active study strategy of working practice questions is associated with improved performance on the United States Medical Licensing Examination (USMLE) Step 1 exam when compared to students who used the passive study strategy of watching educational videos.

Methods

The study used a correlational design. Participants were students from two cohorts in a United States medical school (N=164 and N=163) who completed their first two years and took the USMLE Step 1 exam. Data collected retrospectively included the number of practice questions completed, educational videos watched, Step 1 exam scores, average scores on in-class exams, and scores on the Medical College Admission Test (MCAT).

Results

The number of videos watched was negatively and significantly correlated with the Step 1 score for cohort 2022 (r= -0.294, α=0.01) and cohort 2023 (r= -0.175, α=0.05). The number of practice questions worked was positively and significantly correlated with the Step 1 score for cohort 2022 (r=0.176, α=0.05) and cohort 2023 (r=0.143 though not significant). The number of practice questions was a significant positive predictor of Step 1 score for cohort 2022 (β=0.141, p=0.017) and cohort 2023 (β=0.133, p=0.015). Videos were significant negative predictors for cohort 2023 (β= -0.118, p=0.034).

Conclusions

Answering practice questions appears to be a more effective study method than passively watching videos. Though other studies have supported the use of active learning methods, this study is unique in finding a negative correlation between test scores and the number of educational videos watched. Medical students should be urged to make the most effective use of study time by incorporating working practice questions and limiting watching educational videos.

## Introduction

Helping medical students who are struggling with academic difficulties is a challenging task for medical educators. As of yet, there is not a clear and consistent approach in the literature on how to help struggling medical students [1-3]. Reasons for students' underperformance vary and might include poor study habits, learning disorders, attention deficits, mental health issues, life stressors, and others [4].

Challenges for medical students include performing well on standardized exams within their coursework and on board exams required for academic progression. Performance on the United States Medical Licensure Exam (USMLE) Step 1 and Step 2 Clinical Knowledge (CK) board exams can affect students' residency selections and career choices; therefore, a major challenge for medical schools in the United States of America (USA) and Canada is to help students improve course exam scores and performance on the USMLE Step 1 and Step 2 examinations [5-7].

Active study methods, including retrieval practice, spaced repetition, and the use of practice questions and question banks (a pool of practice questions), have been shown to be effective means of improving students' learning and long-term retention [8-11]. Practice questions can specifically identify weak areas, improve students' metacognition and long-term retention, increase their knowledge, and improve their confidence when approaching standardized exams [12-15]. Specifically, recent research has focused on students' use of practice questions to prepare for board exams, including the USMLE Step 2 CK examination [16-18].

Various educational videos from different sources have been shown to be the most popular e-learning modality [19]. Interestingly, high-performing learners may be more likely to use active learning strategies, while struggling students may avoid answering practice questions and instead use passive study methods such as reading, highlighting, and watching instructional videos [20,21]. The purpose of this study was to investigate if students' use of the recommended active study strategy of using practice questions was associated with improved performance on the USMLE Step 1 board exam compared with students using the passive strategy of watching educational videos. This study adds to the existing literature by focusing specifically on a comparison of question banks and video watching and by analyzing student usage data as opposed to surveying medical students about their study behaviors.

## Materials and methods

Setting

The University of Arkansas for Medical Sciences (UAMS) College of Medicine (COM) is a four-year public medical school located in the southern region of the United States of America (USA). The curriculum is organized into two pre-clinical years followed by two clinical years [22].

Data indicated that students in UAMS COM who underperformed in their first and second years of medical school had a higher failure rate on the USMLE Step 1 exam. Based on data showing that academic coaching and peer tutoring helped struggling students, the UAMS COM implemented a policy in 2019 requiring students who score 75% or less on three or more major course exams to meet with a learning specialist in the Student Success Center and develop an individualized study plan to support academic success. A student's individualized study plan encourages evidence-based active study methods and the use of practice questions and may include regular academic coaching and peer tutoring.

Study design and participants

The study, which used a correlational design, was approved by the University of Arkansas for Medical Sciences Institutional Review Board for Clinical Research Administration (CLARA) as not human subjects research (IRB number 273939). Study participants were medical students from two cohorts, the cohort graduating in 2022 (N=164) and the cohort graduating in 2023 (N=163), who successfully completed their first and second (pre-clinical) years and took the USMLE Step 1 exam.

These two cohorts had similar demographic characteristics and similar scores on the Medical College Admission Test (MCAT) entry exam. Differences between the cohorts included the fact that the 2022 students faced random delays in taking their Step 1 exams due to COVID-related issues, and the 2023 students attended their second year of medical school entirely online (Table 1).

**Table 1:** Cohort 2022 (N=164) descriptive statistics USMLE - United States Medical Licensing Examination, MCAT - Medical College Admission Test

Cohort 2022	Number (N)	Mean (M)	Standard deviation (SD)	Minimum	Maximum
First-year average of in-class exams	164	88.70	4.85	75	98
Second-year average of in-class exams	164	87.29	4.95	72	99
Number of practice questions completed	164	853.62	632.63	0	2461
Number of videos watched	164	144.68	207.14	0	1077
USMLE Step 1 score	164	228.16	20.21	163	275
MCAT score	162	508.16	6.23	494	523

The 2023 students had similar characteristics, except they attended their second year of medical school entirely online due to the COVID-19 pandemic (Table 2).

**Table 2:** Cohort 2023 (N=163) descriptive statistics USMLE - United States Medical Licensing Examination, MCAT - Medical College Admission Test

Cohort 2023	Number (N)	Mean (M)	Standard deviation (SD)	Minimum	Maximum
First-year average of in-class exams	163	88.54	4.95	74	97
Second-year average of in-class exams	163	82.87	6.37	68	98
Number of practice questions completed	163	954.97	395.72	390	2417
Number of videos watched	163	119.02	168.46	0	1013
USMLE Step 1 score	163	222.00	18.62	180	259
MCAT score	163	507.15	5.24	495	522

Study materials

Students had access to the USMLE Rx commercial practice materials, which included a question bank of numerous Step 1 practice questions with case backgrounds and also educational videos from the same commercial product.

Data collection

Data were collected retrospectively and included: the number of USMLE Rx practice questions completed and educational videos watched by students during the first two years of medical school, scores on the USMLE Step 1 exam, average scores on in-class exams during the first and second years of medical school, and scores on the Medical College Admission Test (MCAT).

Analysis

Statistical analysis was performed using SPSS (IBM Inc., Armonk, New York). Data were analyzed using Pearson correlations to assess associations between Step 1 scores and the number of questions completed and videos watched. Multiple linear regression was used to assess the prediction of the dependent variable, USMLE Step 1 score, by a set of independent variables (predictors): MCAT scores, first-year in-class exam scores, second-year in-class exam scores, number of questions completed, and number of videos watched. All probability (p) values were two-sided, with the alpha level set at 0.05.

## Results

Descriptive statistics of cohorts and correlations

Cohorts 2022 and 2023 were similar in terms of first-year in-class exam scores, the number of practice questions completed, videos watched, and MCAT scores (Table 1) but differed in second-year in-class exam scores and USMLE Step 1 scores. There is a large variation among the number of practice questions students worked on and the number of videos they watched.

Pearson correlations for cohort 2022 among the first- and second-year averages of in-class exams, number of practice questions completed, number of videos watched, and USMLE Step 1 score are presented in Table 3.

**Table 3:** Correlational matrix for variables, cohort 2022 USMLE - United States Medical Licensing Examination *Statistically significant at alpha=0.05 **Statistically significant at alpha=0.01

Variables	1	2	3	4	5
First-year average of in-class exams	1				
Second-year average of in-class exams	0.835**	1			
Number of practice questions completed	0.05	0.11	1		
Number of videos watched	-0.36**	-0.278**	0.284**	1	
USMLE Step 1 score	0.653**	0.681**	0.176*	-0.294**	1

Pearson correlations for cohort 2023 among first- and second-year averages of in-class exams, number of practice questions completed, number of videos watched, and USMLE Step 1 score are presented in Table 4. 

**Table 4:** Correlational matrix for variables, cohort 2023 USMLE - United States Medical Licensing Examination *Statistically significant at alpha=0.05 **Statistically significant at alpha=0.01

Variables	1	2	3	4	5
First-year average of in-class exams	1				
Second-year of in-class exams	0.834**	1			
Number of practice questions completed	0.041	0.07	1		
Number of videos watched	-0.107	-0.074	0.247**	1	
USMLE Step 1 score	0.65**	0.706**	0.143	-0.175*	1

For both cohorts, working on more practice questions was associated with higher Step 1 scores, while watching more videos was associated with lower Step 1 scores. For cohort 2022, the number of practice questions completed was positively correlated with Step 1 score r=.176, α=.05, and the number of videos watched was negatively correlated with Step 1 score r = -.294, α=.01. For cohort 2023, the number of practice questions completed was positively, though not significantly, correlated with Step 1 score r=.143, and the number of videos watched was negatively correlated with Step 1 score, r= -.175, α=.05.

Multiple linear regression

A simultaneous multiple regression was performed to investigate the contribution of each independent variable (MCAT scores, first- and second-year averages of in-class exams, number of questions completed, and videos watched) in predicting the dependent variable (Step 1 score) for cohorts 2022 and 2023. For both cohorts, the model explained a statistically significant amount of variance in the Step 1 score. For cohort 2022, R2=.537, F(5,156)=36.17, p<.001, and for cohort 2023, R 2=.571, F(5,157)=41.81, p<.001.

For cohort 2022, the second-year average of in-class exams was the strongest predictor of the Step 1 score (β=.404, p<.001). MCAT was the second predictor (β=.186, p=.004), and the third predictor was the number of questions worked (β=.141, p=.017). Watching videos was a negative predictor. (β= -.098, p=.129) but not statistically significant (Table 5).

**Table 5:** Results of simultaneous multiple regression for predicting the Step 1 score for cohort 2022, N=164 MCAT - Medical College Admission Test Dependent variable: United States Medical Licensing Examination (USMLE) Step 1 score

Variables	Unstandardized beta (B)	Standard error (SE)	Standardized beta (β)	95% confidence interval (CI)		Probability (p)
				Lower limit (LL)	Upper limit (UL)	
First-year average of in-class exams	0.755	0.426	0.182	-0.087	1.597	0.087
Second-year average of in-class exams	1.64	0.405	0.404	0.841	2.433	<0.001
Number of practice questions	0.004	0.002	0.141	0.001	0.008	0.017
Number of videos watched	-0.009	0.006	-0.098	-0.022	0.003	0.129
MCAT	0.599	0.205	0.186	0.194	1.004	0.004

For cohort 2023, the second-year average of in-class exams was the strongest predictor of Step 1 score (β=.517, p<.001). MCAT was the second predictor (β=.184, p=.001), and the third predictor was the number of questions worked (β=.133, p=.015). Watching videos was a negative predictor and was statistically significant for this cohort (β=-.118, p=.034) (Table 6).

**Table 6:** Results of simultaneous multiple regression for predicting the Step 1 score for cohort 2023, N=163 MCAT - Medical College Admission Test Dependent variable: United States Medical Licensing Examination (USMLE) Step 1 score

Variables	Unstandardized beta (B)	Standard error (SE)	Standardized beta (β)	95% confidence interval (CI)		Probability (p)
				Lower limit (LL)	Upper limit (UL)	
First-year average of in-class exams	0.558	0.36	0.148	-0.153	1.269	0.123
Second-year average of in-class exams	1.512	0.278	0.517	0.963	2.06	<0.001
Number of practice questions	0.006	0.003	0.133	0.001	0.011	0.015
Number of videos watched	-0.013	0.006	-0.118	-0.025	-0.001	0.034
MCAT	0.656	0.197	0.184	0.266	1.046	0.001

In summary, for both cohorts, the second-year average of in-class exams was the strongest predictor of Step 1 scores. MCAT was the second predictor of Step 1 scores for both cohorts. The number of practice questions was a significant positive predictor of Step 1 scores. Videos were significant negative predictors for cohort 2023; for cohort 2022, videos were also a negative predictor but not statistically significant. First-year averages of in-class exams were not statistically significant predictors for Step 1 for both cohorts.

## Discussion

The purpose of this study was to investigate if students utilizing the active study strategy of using practice questions was associated with earning higher scores on the USMLE Step 1 board exam compared with students using the passive study strategy of watching educational videos. The findings were that the active study strategy of working practice questions was associated with higher Step 1 scores, and passively watching educational videos was associated with lower Step 1 scores.

Other studies have addressed the importance of retrieval practice in preparing for exams [8,9,11,12,15,21] and the need for a support system for medical students, especially those who are struggling [1-5]. This study focused on a comparison of Step 1 performance and the students' usage of question banks and educational videos.

The findings from this study can help inform study preparation for any medical knowledge tests, even though other medical schools may use different board exams. This study looked specifically at Step 1 board exam scores, which were reported as three-digit scores until January 2022. Step 1 is now reported as pass/fail only, but the importance of maximizing test performance remains [6,17,18].

Using a question bank with high-quality questions prompts cognitive retrieval practice and students' critical thinking, which are important for all medical students. The quality of questions in a question bank is vital to helping students prepare for in-class exams and board exams. Whether questions are designed by faculty or are in a commercial question bank, of chief importance is that the questions are well-written, constructively peer-reviewed, and contain content that prompts critical thinking and analysis [20].

In comparison, educational video content varies in that some videos contain optional self-assessments while others do not. Additionally, it is not possible to know if or how students are engaging in the video material. Furthermore, passive listening and watching may not offer the same learning advantage as active study methods such as working questions [20]. It may be that struggling students do not feel ready to quiz themselves with practice questions, thus choosing to watch educational videos before working on practice questions. The resulting lack of application of concepts does not support students' long-term retention [4].

Results of the multiple regression analysis indicate that the strongest predictor of the Step 1 score is students' performance on the second-year in-class exams, which suggests that engaging students in the curriculum should be prioritized. Not surprisingly, the MCAT score is a predictor of the Step 1 score, as previous preparation and test-taking skills may support the transition to medical school. The number of practice questions worked is also a positive predictor of Step 1 scores, which suggests that students should be encouraged to use active study strategies such as working questions. The number of informational videos watched was a negative predictor of Step 1 scores, which suggests that students should be urged to limit watching videos and instead opt for more active study strategies.

One limitation regarding data on students watching videos is that there is little research about students' attention and engagement while videos are playing. Additionally, little is known about what other resources students may be using to study beyond those recommended by the medical school.

## Conclusions

A strength of the study was the specific focus on active versus passive study strategies and their potential impact on preparation for a critically important board exam. Additionally, the study habits were not from self-reported survey results but were observed by student usage data of resources that were equitably available to all students. It should be noted that students' educational experiences were impacted by the COVID-19 pandemic, and future research may investigate the long-term effects of educational disruption, physical illness, and mental stressors due to the pandemic. 

The results of this study support the premise that answering practice questions was an effective study method, but add a notable distinction regarding active versus passive study methods employed by students. Unique to this study was the finding that watching educational videos had a negative correlation with Step 1 scores. Medical students should be urged to make the most effective use of study time by limiting passively watching educational videos and instead actively incorporating the regular use of practice questions into their study. 
